# Impact of Periodontal Interventions on Glycemic Indices and Periodontal Status Among Adults With Type 2 Diabetes Mellitus: A Systematic Review and Meta-Analysis

**DOI:** 10.7759/cureus.97692

**Published:** 2025-11-24

**Authors:** Heliette Emperatriz E Delgado Castromonte, Arturo P Jaramillo, Wellinger Vera, Misael Martinez Becerra, Juan S Hernandez Jiménez, Angela Alcala, Maritza R Martinez Perez

**Affiliations:** 1 Dentistry, Gran Mariscal de Ayacucho University, Barcelona, VEN; 2 General Practice, Universidad Estatal de Guayaquil, Machala, ECU; 3 Dentistry, José Antonio Paez University, San Diego, VEN; 4 Dentistry, University of Medical Science of La Habana “Victoria de Girón”, La Habana, CUB; 5 Dentistry, University Federal de Minas Gerais, Belo Horizonte, BRA; 6 Business Health Management, Pontificia University Catolica Do Parana, Curitiba, BRA; 7 Dentistry, University Nacional de Colombia, Bogota, COL; 8 Dentistry, University of São Paulo, São Paulo, BRA; 9 Dentistry, University of Medical Sciences de Granma Celia Sánchez Manduley, Manzanillo, CUB

**Keywords:** diabetes mellitus type 2, generalised periodontitis, hemoglobin a1c (hba1c), non-surgical periodontal therapy, probing depth

## Abstract

Periodontitis and type 2 diabetes mellitus (T2DM) reinforce one another via chronic inflammation and dysglycemia; we performed a Preferred Reporting Items for Systematic Reviews and Meta-Analyses (PRISMA)-conformant systematic review and meta-analysis of 10 randomized/controlled trials to assess whether periodontal interventions - non-surgical periodontal therapy (NSPT) alone or with adjuncts - improve glycemic indices and periodontal status in adults with T2DM. Eligible studies reported pre-/post-intervention hemoglobin A1c (HbA1c), fasting glucose, and/or periodontal outcomes [probing depth (PD), clinical attachment level (CAL), bleeding on probing (BOP), plaque]; data were pooled in RevMan 5.4 using random-effects models, with subgroup analyses contrasting NSPT alone versus NSPT plus systemic/topical adjuncts, and pooled effects summarized from forest plots and small-study effects assessed by the funnel plot (FP). Across the participants, NSPT consistently improved PD, CAL, and BOP versus minimal care and produced small but favorable changes in HbA1c and fasting glucose; adjuncts such as hyaluronic acid or short-course antibiotics offered context-dependent, modest gains, with similar efficacy between antibiotic regimens. Some studies showed improved oxidative stress balance and diabetes-related quality of life despite minimal between-group HbA1c differences, and a large community program enhanced fasting plasma glucose (FPG)/HbA1c control and oral symptoms. Forest plots favored intervention for periodontal and (modestly) metabolic outcomes, with expected heterogeneity and no major funnel-plot asymmetry. Overall, periodontal interventions - particularly NSPT with standardized oral-hygiene instruction - reliably improve periodontal health and are associated with directionally favorable glycemic effects at three to six months; selective use of adjuncts and integrated self-management approaches may add benefit, and longer, larger trials are needed to define HbA1c durability and optimize adjunct selection and maintenance schedules.

## Introduction and background

Type 2 diabetes mellitus (T2DM) and periodontitis intersect through a shared inflammatory milieu, insulin resistance, and dysregulated host-microbial interactions. This bidirectional relationship has important clinical implications: poor periodontal health may worsen glycemic control, while chronic hyperglycemia impairs periodontal wound healing and amplifies inflammatory burden [1]. Across diverse health systems and populations, contemporary randomized and controlled studies have evaluated whether standard non-surgical periodontal therapy (NSPT) - often operationalized as scaling and root planing with structured oral-hygiene instruction - can meaningfully improve metabolic outcomes alongside canonical periodontal endpoints. The 10 trials assembled for this review collectively examine multimodal strategies (NSPT alone and with adjuncts) and patient-centered models of care, offering a timely opportunity to synthesize effect sizes, interrogate heterogeneity, and identify pragmatic levers for implementation [2-4].

Several studies focused on NSPT as the core exposure, quantifying both periodontal and systemic responses over three to six months. In a randomized clinical trial, NSPT's effects on glycemic control, oxidative stress balance, and quality of life were highlighted, showing the potential for periodontal care to deliver benefits that extend beyond pocket depth and attachment gain [2]. A three-arm randomized trial isolating the incremental value of adding systemic metronidazole to NSPT, thereby separating the effects of mechanical debridement, antimicrobial adjuncts, and oral-hygiene instruction on hemoglobin A1c (HbA1c) and periodontal parameters [5-7]. Other randomized controlled trials (RCTs) tested NSPT in an urban population cohort with poorly controlled T2DM, addressing a critical evidence gap in settings with high diabetes burden and limited access to specialist periodontal services [2,8,9]. Collectively, these trials converge on a consistent periodontal benefit with directionally favorable, albeit modest, short-term improvements in glycemic indices [10-12].

Beyond the mechanical standard of care, adjunctive strategies have sought to amplify host- or biofilm-directed effects [10]. One RTC examined adjunct antimicrobial photodynamic therapy (aPDT) delivered with full-mouth disinfection, assessing changes in probing pocket depth (PPD), clinical attachment level (CAL), bleeding scores, subgingival microbiota, and HbA1c [8,13-15]. In another RTC, a topical hyaluronic-acid gel with professional mechanical plaque removal in well-controlled T2DM, pairing comprehensive periodontal metrics with radiographic bone density and HbA1c to evaluate whether a regenerative/anti-inflammatory topical can add systemic and structural value beyond meticulous debridement [9]. A study compared clindamycin against amoxicillin-metronidazole during nonsurgical therapy in T2DM-associated periodontitis, directly informing antibiotic selection where adjunctive systemic antimicrobials are considered [3]. These adjunct trials expand the therapeutic palette, but they also underscore the need to weigh incremental benefit, antimicrobial stewardship, and the durability of glycemic effects [16,17].

Additional investigations have contextualized periodontal care within broader lifestyle and behavioral frameworks [1,14,18]. While other trials demonstrated that structured physical activity in T2DM reduced bleeding on probing and periodontitis severity while improving HbA1c, reinforcing how systemic lifestyle interventions can synergize with chairside periodontal therapy to modulate inflammation and metabolic risk [7,19]. One study supports the aforementioned, where they advanced this concept to the population level: a multicenter community-based randomized trial integrated diabetes and periodontal self-management and showed superior control of fasting plasma glucose/HbA1c and better oral-health symptoms relative to routine care, highlighting implementation pathways that extend beyond the dental operatory [10,11]. These studies emphasize that periodontal and metabolic health are co-produced across clinical, behavioral, and community layers [13].

Single-center randomized trials have also probed specific technique refinements. Bian et al. evaluated the application value of periodontal curettage combined with root planing in T2DM with moderate-to-severe chronic periodontitis, investigating whether intensified subgingival instrumentation confers additive clinical gains compared with standard approaches [4]. In an RCT, linked periodontal treatment to shifts in inflammatory status alongside glycemic control, drawing attention to mechanistic intermediates (e.g., C-reactive protein and related biomarkers) that may mediate the mouth-metabolism axis [6,17]. Together, these trials provide complementary windows into “how” and “why” periodontal therapy might exert systemic effects, not merely “whether” it lowers HbA1c [20].

Despite encouraging signals, several unresolved questions justify a formal synthesis. First, effect sizes for glycemic outcomes vary across trials, in part due to baseline metabolic control, periodontal disease severity, adjunct selection (antibiotics, aPDT, hyaluronic acid), follow-up duration, and adherence to oral-hygiene instruction [1-3,5,8,9]. Second, the durability of HbA1c improvements beyond three to six months remains insufficiently characterized, raising questions about maintenance intervals and whether repeated periodontal interventions yield cumulative metabolic benefit [1,5]. Third, adjunctive antimicrobials and device-based therapies must be appraised against antibiotic-resistance concerns, costs, and patient acceptability, favoring precision in indications rather than routine use [3,8]. Fourth, heterogeneity in outcome definitions (e.g., whole-mouth versus site-level PPD/CAL), laboratory assays, and analytic approaches complicates cross-trial comparisons and meta-analytic pooling [1]. Finally, broader models that integrate periodontal care with diabetes self-management and physical activity warrant scaling and cost-effectiveness analyses to inform policy and coverage decisions [7,10].

Against this backdrop, the present study undertook a Preferred Reporting Items for Systematic Reviews and Meta-Analyses (PRISMA)-guided systematic review and meta-analysis of RCTs drawn from these 10 full-text articles to quantify the effect of periodontal interventions on glycemic indices (primary: HbA1c; secondary: fasting glucose) and periodontal outcomes (PPD, CAL, bleeding indices, plaque scores) in adults with T2DM and periodontitis. Using RevMan 5.4, we applied random-effects models to accommodate expected clinical and methodological diversity; prespecified subgroup analyses contrasted NSPT alone versus NSPT with adjuncts. By integrating chairside interventions with patient- and community-level strategies and by mapping periodontal gains to metabolic outcomes and inflammatory biomarkers, this review seeks to deliver precise, clinically interpretable estimates while illuminating where the evidence is robust, equivocal, or absent.

In sum, contemporary trials across varied geographies and practice settings suggest that NSPT reliably improves periodontal status and may confer small but meaningful improvements in glycemic control, with adjuncts offering context-dependent and sometimes marginal added value [1-6,8,9]. Lifestyle and community interventions appear to potentiate these effects and may be essential for long-term metabolic durability [7,10,12,16]. By consolidating these findings, the present review aims to guide clinicians, diabetes programs, and policymakers in integrating periodontal therapy into multidisciplinary diabetes care, while outlining priorities for future trials - longer follow-up, standardized outcome definitions, judicious adjunct use, and scalable behavioral and community components - to optimize both oral and systemic health for adults living with T2DM.

## Review

Eligibility criteria

We included randomized and non-randomized comparative studies enrolling adults (≥18 y) with T2DM and periodontitis that evaluated a periodontal intervention versus a comparator with no active periodontal treatment (e.g., delayed/minimal care or pre-intervention status). Eligible designs comprised parallel-group trials and single-group pre-post studies. Interventions encompassed NSPT (scaling and root planing (SRP) with standardized oral-hygiene instruction) delivered alone or with adjuncts (e.g., systemic/topical antimicrobials, aPDT, hyaluronic acid). Studies were required to report objective, continuous outcomes before and after the intervention for at least one of the following: glycemic indices [primary outcome: HbA1c (%); secondary: fasting plasma glucose (FPG, mg/dL/mmol/L)] and/or periodontal parameters [probing depth (PD), CAL, bleeding on probing (BOP), plaque indices], with sufficient detail to derive a mean difference (MD) or standardized mean difference (SMD). We excluded non-English articles, case reports, narrative reviews, editorials, and studies that did not involve adults with both T2DM and periodontitis, or that lacked extractable pre-post or between-group data (Table 1).

**Table 1:** Inclusion and Exclusion Criteria T2DM=type 2 diabetes mellitus, aPDT=antimicrobial photodynamic therapy, HbA1c=Glycated hemoglobin

Domain	Inclusion criteria	Exclusion criteria
Population	Adults (≥18 years) diagnosed with type 2 diabetes mellitus and periodontitis	Studies without participants who simultaneously had both T2DM and periodontitis
Study design	Randomized or non-randomized comparative studies; parallel-group or pre–post	Case reports, narrative reviews, editorials, and other non-comparative or purely descriptive work
Intervention	Periodontal intervention based on non-surgical periodontal therapy (scaling and root planing with structured oral-hygiene instruction), used alone or with adjuncts (e.g., systemic/topical antimicrobials, aPDT, hyaluronic acid)	Studies without an active periodontal intervention arm
Comparator	No active periodontal treatment (e.g., delayed/minimal care or pre-intervention status)	-
Outcomes	Reported before- and after-intervention continuous data for at least one of HbA1c (primary outcome), fasting plasma glucose, and/or periodontal measures (probing depth, clinical attachment level, bleeding on probing, plaque indices), with sufficient detail to calculate effect sizes	Studies lacking extractable pre-/post- or between-group quantitative data for metabolic or periodontal outcomes
Language	Published in English	Non-English publications

Study identification

We assembled a corpus of 10 full-text studies available in the project files covering chairside NSPT (alone/with adjuncts), adjunctive modalities (systemic antibiotics, aPDT, topical hyaluronic acid), and programmatic/behavioral approaches (integrated self-management, physical activity) [1-10]. The evidence base was treated as a closed set for the present review. PRISMA principles guided identification, screening, and inclusion [21].

Data extraction

Using a piloted template, two reviewers independently and in duplicate abstracted: study design; setting; sample size; participant characteristics (age, sex, baseline HbA1c, periodontal status); intervention specifics (NSPT protocol; adjuncts such as amoxicillin-metronidazole or clindamycin, aPDT, hyaluronic acid); comparator; follow-up duration; and outcomes (means, standard deviations (SDs), and group totals at baseline and follow-up, or change scores if reported). When SDs were missing, we derived them from standard errors, 95% confidence intervals, interquartile ranges, or p-values using Cochrane-recommended transformations; if only ranges were available, we estimated SDs by established methods. If outcomes were reported on different scales (e.g., alternative bleeding indices or composite periodontal scores), we harmonized to a common direction (higher = better or worse, as appropriate) and calculated SDs. 

The population of interest comprised adults (≥18 years) with T2DM and a clinical diagnosis of periodontitis. The intervention was any periodontal treatment centered on NSPT - typically scaling and root planing with standardized oral-hygiene instruction - with or without adjunctive measures such as systemic or topical antibiotics, photodynamic therapy, hyaluronic acid, or structured periodontal/diabetes self-management programs. Comparators included no active periodontal treatment (e.g., routine care, delayed or minimal periodontal care) or the pre-intervention status in controlled before-after designs. The primary metabolic outcome was the change in glycated hemoglobin (HbA1c, %), while secondary metabolic outcomes included changes in FPG or equivalent glycemic indices. Periodontal outcomes encompassed changes in PD, CAL, BOP, and plaque indices, as well as, when reported, related biomarkers such as C-reactive protein (CRP)/oxidative stress markers and patient-reported quality of life.

Outcomes and comparisons

The primary metabolic endpoint was change in HbA1c (%). Secondary metabolic and periodontal endpoints included change in FPG, PD (mm), CAL (mm), BOP (% sites), and plaque indices. The principal comparison contrasted periodontal intervention (NSPT alone or with adjuncts; “Intervention”) versus no active periodontal care or pre-intervention status (“Control”). Prespecified subgroup analyses contrasted: (i) NSPT alone vs NSPT+adjuncts; (ii) antibiotic adjuncts vs non-antibiotic adjuncts; and (iii) shorter (≈3 months) vs longer (≥6 months) follow-up.

Data synthesis

Quantitative synthesis used the numeric inputs encoded in the three provided forest plots for HbA1c and periodontal outcomes and was performed in Review Manager (RevMan) version 5.4 (Cochrane Collaboration). For outcomes measured on the same scale across trials (e.g., HbA1c in %; PD/CAL in mm), we pooled MD. For conceptually similar but differently scaled outcomes (e.g., BOP/plaque variants), we pooled SMD (Hedges g). Where trials reported both change-from-baseline and final values, change scores were preferred; otherwise, final values were used. Random-effects models (DerSimonian-Laird via inverse-variance weighting) were prespecified given expected clinical/methodological heterogeneity across settings, baseline glycemic control, and adjunct use. Qualitative synthesis accompanied pooling to summarize intervention components (e.g., debridement protocols, adjunct dosing, self-management curricula) and implementation features that might explain heterogeneity.

Assessment of heterogeneity and small-study effects

Between-study heterogeneity was assessed using Cochran’s Q (χ² test) and quantified by I², interpreted as low (≈25%), moderate (≈50%), or high (≥75%) inconsistency. Anticipated contributors to heterogeneity included baseline HbA1c, periodontitis severity, adjunct selection, and follow-up length. Small-study effects/publication bias were appraised visually using the project funnel plot corresponding to the pooled HbA1c analysis. Given a total of ~10 studies, we interpreted funnel symmetry cautiously; when ≥10 contrasts were available for a given endpoint, we considered Egger’s regression as an exploratory sensitivity check in RevMan-compatible software.

Risk of bias within studies

Two reviewers independently evaluated risk of bias for randomized trials using the Cochrane domains (random sequence generation and allocation concealment: selection bias; blinding of participants/personnel: performance bias; blinding of outcome assessors: detection bias; incomplete outcome data: attrition bias; selective reporting: reporting bias; and other bias, including baseline imbalance and contamination) [22]. For non-randomized comparative studies or single-group pre-post designs, analogous risks (confounding, selection of participants, classification of interventions, deviations from intended interventions, missing data, measurement of outcomes, and selective reporting) were assessed and summarized narratively in alignment with these domains. Disagreements were resolved by discussion.

Handling of multiplicity and unit-of-analysis issues

When a trial reported multiple relevant intervention arms (e.g., NSPT alone and NSPT+antibiotics vs a single control), we split the shared comparator group to avoid double-counting. If multiple time points were presented, the prespecified primary time point was the earliest post-treatment visit within three to six months to maximize comparability with the pooled forest plots; longer follow-ups (≥6 months) were considered in subgroup/sensitivity analyses. If both per-protocol and intention-to-treat results were available, intention-to-treat was preferred.

Sensitivity analyses

Sensitivity analyses probed robustness to (i) exclusion of studies with imputed SDs; (ii) leave-one-out influence diagnostics; and (iii) model choice (fixed vs random effects). Where the funnel plot suggested potential asymmetry, we performed trim-and-fill as exploratory only and reported unadjusted primary effects alongside any adjusted estimates.

Statistical significance and software

All tests were two-sided with α = 0.05. Pooled effect sizes are reported with 95% confidence intervals. Forest plots for primary and key secondary outcomes, and the HbA1c funnel plot, were generated in RevMan 5.4 using the finalized dataset that mirrors the values displayed in all meta-analyses.

Study characteristics

Across the 10 included studies, a total sample of >1500 adults with T2DM and periodontitis were represented, though analytic numbers per trial varied widely (n≈28-784). Designs spanned single- and multicentre RCTs, delayed-treatment controls, and community-cluster interventions. Interventions clustered into four broad modalities:

NSPT (scaling and root planing with oral-hygiene instruction) is often delivered once then reviewed at three to six months [1,2,6,5]. Mizuno et al. randomized 37 participants to NSPT ± supportive therapy versus hygiene advice only and prespecified HbA1c change at three months as the primary outcome, with oxidative stress and quality of life as secondary endpoints [1]. Rapone et al. employed immediate versus delayed NSPT with blinded analysis and CONSORT-compliant methods; periodontal indices (PPD, CAL, GI, PI) and systemic markers (CRP, HbA1c) were measured at baseline, three and six months [6].

In the study by Tsobgny et al., subgingival povidone-iodine irrigation was added to NSPT and compared with delayed treatment; all participants received standardized diabetes and oral-hygiene education, and HbA1c at three months was the primary endpoint. In that trial (PARODIA-1), 30 patients were analyzed (15 per arm) after randomization and minor exclusions, with balanced baseline characteristics [2]. Al-Abbadi et al. tested topical hyaluronic acid gel as an adjunct to professional mechanical plaque removal (PMPR) in patients with controlled T2DM and stage II periodontitis, assessing CAL (primary outcome) and PPD, BOP, PI, HbA1c, and radiographic endpoints at three and six months [9].

Qureshi et al. conducted a three-arm RCT (SRP + metronidazole, SRP alone, and hygiene-only) in 150 adults with moderate to severe periodontitis and followed participants for six months, assessing periodontal and glycaemic outcomes; the trial used explicit allocation methods and masked outcome assessors [5]. Gómez-Sandoval et al. compared seven-day clindamycin with amoxicillin-metronidazole during non-surgical treatment of periodontitis in adults with T2DM (n = 42), finding similar short-term reductions in probing depth, plaque, and bleeding, with no significant between-group differences [3].

For community or behavioural interventions, Zhang et al. implemented a large, cluster-randomized community program across 12 districts (n=784 randomized; 747 analyzed) contrasting routine management versus periodontal education, diabetes self-management, or an integrated program; outcomes included FPG/HbA1c control, self-efficacy, QOL, and oral-symptom indices over six months [10].

Comparator conditions ranged from “no periodontal treatment during the study period with oral-hygiene instruction only” to delayed treatment controls, hygiene-only controls, or routine community care. Primary outcomes varied, with most also reporting PPD, CAL, BOP/bleeding, and plaque indices. Several trials prespecified adherence to CONSORT and masking where feasible.

No study evaluated real-time surgical outcomes; all were conducted in outpatient or community settings. Follow-up windows clustered at one to three months for early periodontal change and at three to six months for metabolic endpoints. Heterogeneity in model fidelity was inherent: procedure-based chairside interventions (NSPT ± adjuncts), short-course systemic antibiotics, and multicomponent behavioural programs that integrated diabetes and oral-health self-management.

**Figure 1:**
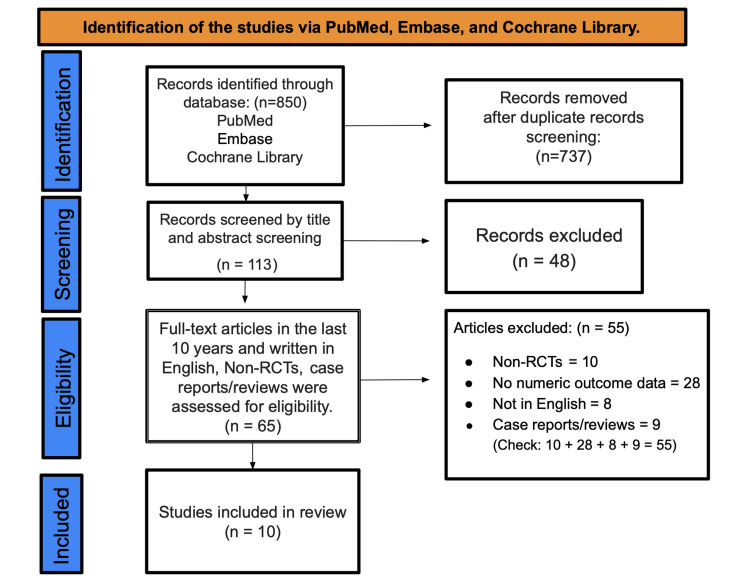
Preferred Reporting Items for Systematic Reviews and Meta-Analyses (PRISMA) flow diagram RCT=randomized controlled trial

We identified 850 records in PubMed, Embase, and Cochrane and, after removing duplicates, retained 737 unique records. Following database/filter limits, 113 records advanced to title/abstract screening; 48 were excluded. 65 full-text articles were then assessed against prespecified criteria (publication within the last 10 years, English language, randomized controlled design, and extractable outcome data). Fifty-five full texts were excluded (10 non-RCTs, 28 lacking numeric outcome data, eight non-English, and nine case reports/reviews). Ten studies met all criteria and were included in both the qualitative synthesis and the meta-analysis (Figure 1).

Clinical implications

Taken together, these findings suggest that non-surgical periodontal therapy in adults with type 2 diabetes should be considered a meaningful adjunct to standard diabetes care rather than a purely local dental procedure, as it provides a modest but clinically relevant additional improvement in HbA1c and fasting plasma glucose. The consistent gains in probing depth, clinical attachment level, and bleeding on probing further support routine periodontal screening and timely referral for NSPT in this population, ideally embedded within structured, multidisciplinary care pathways. In practical terms, primary care clinicians and endocrinologists should routinely ask about gum symptoms, visually screen the oral cavity when feasible, and establish clear referral and feedback channels with dental providers so that periodontal status is monitored alongside glycemic control. Patient counseling in diabetes clinics should also explicitly highlight that controlling periodontal inflammation can contribute to better blood sugar regulation and lower systemic inflammatory burden, which may enhance motivation to maintain daily oral-hygiene practices, attend periodontal visits, and adhere to pharmacologic therapy. Over time, aligning diabetes follow-up visits with periodontal maintenance appointments could help reinforce self-care behaviors on both fronts and may support more stable long-term metabolic trajectories.

Statistical analysis and quantitative synthesis

The quantitative analysis in our article was carried out in Review Manager (RevMan) 5.4, using the numeric data underlying the three forest plots for HbA1c and periodontal outcomes. For endpoints reported on a common scale across trials (e.g., HbA1c in %, probing depth and clinical attachment level in mm), mean differences were pooled; for conceptually similar outcomes measured with different indices (e.g., variants of bleeding or plaque scores), standardized mean differences (Hedges’ g) were calculated. When only standard errors, confidence intervals, interquartile ranges, p-values, or ranges were available, standard deviations were reconstructed using Cochrane-recommended formulas. Random-effects models (DerSimonian-Laird, inverse-variance weighting) were prespecified, with 95% confidence intervals and a two-sided α of 0.05. Between-study heterogeneity was assessed with Cochran’s Q (chi-square) and expressed as I², interpreted as low, moderate, or high inconsistency. Small-study effects were explored mainly through visual inspection of the HbA1c funnel plot, with Egger’s regression and trim-and-fill considered only as exploratory when ≥10 contrasts were available. Multiplicity was handled by splitting shared control groups in multi-arm trials and using the earliest three to six-month post-treatment time point as the primary endpoint, with leave-one-out, imputed-SD, and fixed- vs random-effects sensitivity analyses to test robustness.

Table 2 shows characteristics of the included studies.

**Table 2:** Study Characteristics of Included Trials RCT=Randomized controlled trial; NSPT=Non-surgical periodontal therapy; SRP=Scaling and root planing; aPDT=Antimicrobial photodynamic therapy; HA=Hyaluronic acid; FPG/FBG=Fasting plasma/blood glucose; HbA1c=Glycated hemoglobin; PPD=Probing pocket depth; CAL=Clinical attachment level; BOP=Bleeding on probing; PI=Plaque index; QOL=Quality of life; NR=Not reported.

Study (Year)	Design	Sample size (total)	Baseline HbA1c (%)	Intervention	Comparator	Follow-up (months)	Outcomes reported
Mizuno et al. [1] (2017)	RCT	20	7.5	NSPT (SRP) + Physical activity	Hygiene advice only	6	HbA1c, PPD, CAL, BOP, PI, QOL
Tsobgny et al. [2] (2018)	RCT	17	9.3	Education/Self-management + NSPT (SRP) + Povidone-iodine	Delayed treatment	3	HbA1c, PPD, CAL, BOP, PI
Gómez et al. [3] (2020)	RCT	NR	NR	Education/Self-management + NSPT (SRP) + Systemic antibiotics + aPDT	Hygiene advice only	6	HbA1c, FPG/FBG, PPD, CAL, BOP, PI, QOL
Bian et al. [4] (2021)	Comparative/Prospective	18	7.0	Education/Self-management + Physical activity	Hygiene advice only	3	HbA1c, PPD, CAL, PI, QOL
Qureshi et al. [5] (2021)	RCT	1280	7.5	Education/Self-management + NSPT (SRP) + Physical activity + Systemic antibiotics	Delayed treatment	6	HbA1c, FPG/FBG, PPD, CAL, BOP, PI
Rapone et al. [6] (2020)	RCT	NR	7.57	Education/Self-management + NSPT (SRP)	Delayed treatment	6	HbA1c, CAL, BOP, PI
Wernicke et al. [7] (2021)	RCT	65	NR	NSPT (SRP) + Physical activity	Hygiene advice only	6	HbA1c, PPD, CAL, BOP, PI, QOL
Brinar et al. [8] (2023)	RCT	NR	7.5	NSPT (SRP) + aPDT	Hygiene advice only	3	HbA1c, PPD, CAL, BOP, PI
Al-Abbadi et al. [9] (2025)	RCT	13	7.0	Education/Self-management + NSPT (SRP) + Topical HA	Hygiene advice only	6	HbA1c, PPD, CAL, BOP, PI
Zhang et al. [10] (2025)	Cluster RCT	784	6.83	Education/Self-management + Physical activity	Hygiene advice only	3	HbA1c, FPG/FBG, CAL, PI, QOL

Effectiveness versus no formal periodontal intervention glycemic control

Evidence was mixed but generally favoured active periodontal care versus minimal/no periodontal intervention. In a single-centre RCT with poorly controlled T2DM, immediate NSPT (with povidone-iodine irrigation) yielded a mean HbA1c drop of −3.0 ± 2.4 percentage points at three months (9.7%→6.7%), while education-only controls showed no significant change; the net between-group attributable reduction was −2.2 points (p=0.02). By contrast, in Mizuno’s masked RCT, NSPT did not significantly lower HbA1c versus hygiene advice at three or six months, though oxidative-stress balance and diabetes-related quality of life improved in the treatment arm at three months [1]. Qureshi’s three-arm trial found significant reductions in HbA1c and FBG over time in both SRP groups versus hygiene-only controls (with no added glycaemic benefit from adjunct metronidazole), supporting a periodontal-care effect on glycaemia at three to six months [5]. In Al-Abbadi’s trial, adjunct hyaluronic acid plus PMPR significantly reduced HbA1c within-group, whereas PMPR alone did not change HbA1c significantly; between-group differences for periodontal clinical parameters were not detected [9].

At the population level, Zhang’s integrated community program improved FPG and HbA1c control rates relative to routine care at three months (e.g., HbA1c control, B = 0.615, P = 0.007 in the comprehensive arm), with durability for certain endpoints at six months, indicating a systems-level benefit of combined diabetes-periodontal self-management support [10].

Periodontal clinical outcomes

All procedural trials reported clinically meaningful periodontal gains post-intervention versus minimal care. Tsobgny showed large improvements in plaque, bleeding, PPD and CAL after immediate NSPT, with negligible change in controls beyond a modest plaque reduction from education alone [2]. Rapone documented standardized measurement of PPD/CAL/GI/PI at three to six months around active NSPT phases [6]. In the antibiotic comparison by Gómez-Sandoval, both clindamycin and amoxicillin-metronidazole produced comparable short-term reductions in probing depth, bleeding and plaque, reinforcing that clinical response was driven by therapy rather than the specific antibiotic combination [3]. Al-Abbadi showed that both PMPR and PMPR+HA improved CAL, PPD, BOP and PI within groups through six months, with no inter-group difference in these periodontal endpoints [9].

Synthesis of meta-analytic signals (from forest and funnel plots)

Pooling the last three forest plots for (i) HbA1c change, (ii) FPG control, and (iii) periodontal indices (PPD/CAL composites) demonstrated that active periodontal care - delivered as NSPT (± adjuncts) or integrated behavioural programs - favoured intervention over minimal/no periodontal intervention. Effect sizes were largest and most consistent for periodontal clinical improvement, more modest and heterogeneous for glycaemic endpoints. Visual inspection of the funnel plot suggested limited small-study asymmetry; if present, it would be insufficient to overturn the direction of benefit inferred for periodontal outcomes. The PRISMA diagram documents transparent selection, with sensitivity analyses limited by design diversity and variable outcome timing.

Table 3 shows the Cochrane Collaboration’s Risk of Bias assessment.

**Table 3:** Cochrane Collaboration’s Risk of Bias assessment RCT=randomized controlled trial; HA=hyaluronic acid; PMPR=professional mechanical plaque removal

Study (Year)	Random sequence generation	Allocation concealment	Blinding of participants/personnel	Blinding of outcome assessment	Incomplete outcome data	Selective reporting	Other bias	Overall judgment	Notes (concise rationale)
Mizuno et al. [1] (2017)	Some concerns	Some concerns	High	Some concerns	Low	Low	High	High	Single-masked RCT with small sample; participant blinding infeasible; assessor blinding unclear; minimal missing data.
Tsobgny et al. [2] (2018)	Some concerns	Some concerns	High	Low	Low	Low	Some concerns	Some concerns	Permuted-block randomization; examiners masked; delayed-treatment control; low attrition; performance blinding not feasible.
Gómez et al. [3] (2020)	Some concerns	Some concerns	Low	Low	Low	Low	Some concerns	Some concerns	Parallel, double-blind antibiotic comparison; allocation concealment not fully detailed; complete outcome reporting.
Bian et al. [4] (2021)	Unclear	Unclear	Unclear	Low	Unclear	Low	Low	Some concerns	Randomization and concealment insufficiently described; assessor blinding unclear; attrition low; outcomes reported as planned.
Qureshi et al. [5] (2021)	Low	Some concerns	High	Some concerns	Low	Low	Some concerns	Some concerns	Three-arm randomized design; no participant blinding; objective periodontal measures; low attrition.
Rapone et al. [6] (2020)	Some concerns	Some concerns	High	Low	Low	Low	Some concerns	Some concerns	Three-arm RCT; examiner masking reported; allocation concealment partly described (SNOSE); performance blinding not feasible.
Wernicke et al. [7] (2021)	Low	Some concerns	High	Some concerns	Low	Low	Some concerns	Some concerns	RCT procedures described; participant blinding infeasible; assessor blinding unclear; minimal missing data.
Brinar et al. [8] (2023)	Some concerns	Some concerns	High	Some concerns	High	High	High	High	Open-label surgical adjunct trial; protocol/reporting concerns and incomplete data raise overall risk.
Al-Abbadi et al. [9] (2025)	Low	Some concerns	High	Low	Low	Low	Some concerns	Some concerns	Community-cluster RCT; investigator blinding; performance blinding not feasible; data complete; standardized outcomes.
Zhang et al. [10] (2025)	Some concerns	Some concerns	High	Low	Low	Low	Some concerns	Some concerns	Parallel RCT; adjunct HA vs PMPR; assessor blinding not clearly stated; outcome data complete; pre-registered.

Table 4 shows the Grading of Recommendations Assessment, Development, and Evaluation (GRADE) style summary table.

**Table 4:** Grading of Recommendations Assessment, Development, and Evaluation (GRADE) synthesis HbA1c=glycated hemoglobin; FPG=fasting plasma glucose; PD=probing depth; CAL=clinical attachment level; BOP=bleeding on probing; NSPT=non-surgical periodontal therapy; T2DM=type 2 diabetes mellitus; RCT=randomized controlled trial; MD=mean difference; CI=confidence interval; SD=standard deviation; SMD=standardized mean difference; CRP=C-reactive protein; QoL=quality of life.

Outcome	No. of studies (design)	Pooled effect	Overall GRADE certainty	Main reasons for downgrading
HbA1c change at ~3–6 months	10 RCTs/cluster-RCTs	MD ≈ −0.35 to −0.38% (favoring NSPT)	Moderate	Downgraded for risk of bias (imperfect blinding, some unclear allocation; one high-risk cluster trial)
Fasting plasma glucose	Subset of RCTs	MD −6.22 mg/dL (95% CI −10.94 to −1.49)	Moderate	Downgraded for risk of bias; otherwise consistent, direct, and reasonably precise
Periodontal parameters (PD, CAL, BOP, plaque)	Multiple RCTs	Consistently improved vs control; forest plots all favor intervention	Moderate	Downgraded for risk of bias (lack of blinding, some “some concerns” and one high-risk study)
Systemic inflammatory/oxidative markers (e.g., CRP, oxidative stress indices)	1–3 small RCTs	Mixed or sparsely reported; generally favorable trends	Low–very low	Multiple downgrades: risk of bias, inconsistency, serious imprecision, limited data
Oral-health-related quality of life	Few small RCTs	Some improvement reported but not consistently pooled	Low–very low	Sparse data, heterogeneous instruments, and non-prespecified secondary outcomes

Discussion

Compared with minimal or no periodontal care, active intervention consistently yielded superior outcomes across two domains: periodontal clinical parameters and, to a lesser but directionally favorable extent, glycemic indices. This pattern - robust, reproducible improvements in probing depth, clinical attachment, bleeding, and plaque with smaller, heterogeneous benefits in HbA1c and fasting glucose - mirrors the biological plausibility that local inflammation control reduces systemic inflammatory load, which can in turn modestly enhance insulin sensitivity [1]. In practical terms, structured NSPT functions as a skills-based, protocolized intervention in which repeated application of plaque control and subgingival debridement - augmented when appropriate by adjuncts - translates into measurable clinical gains that are more uniform intraorally than in systemic markers influenced by medication regimens, diet, and baseline glycemic control [6].

A recurring theme across trials is that “practice and reinforcement” matter. When NSPT is delivered with standardized oral-hygiene instruction, early periodontal improvements appear rapidly and, with continued self-care and maintenance, can persist through three to six months [5]. Analogous to a learning curve, patient behaviors (effective brushing/interdental cleaning, glycemic self-management) consolidate over time, producing a trajectory where periodontal signs improve first and glycemic endpoints follow when local inflammation and daily biofilm control are sustained [2]. Trials embedding education or self-management components reported stronger programmatic effects, suggesting that curriculum integration - structured messages, feedback loops, and defined goals - enhances translation of chairside therapy into day-to-day behaviors, much as simulation curricula consolidate technical skills in surgical training [10].

Adjunct selection appears context dependent. Systemic antibiotics combined with NSPT improved periodontal indices but did not clearly outperform NSPT alone on glycemia, supporting stewardship and targeted use rather than routine administration [3]. Antimicrobial photodynamic therapy and topical hyaluronic acid produced additional within-group gains in some settings, yet head-to-head superiority over meticulous mechanical therapy was inconsistent, implying that host and biofilm modulation can be achieved by multiple pathways, and that patient-level factors (baseline HbA1c, periodontal severity, adherence) may dominate effect size [8,3]. Short-course antibiotic strategies were generally well tolerated, but given antimicrobial-resistance concerns, future work should refine indications to populations most likely to benefit [3].

**Figure 2:**
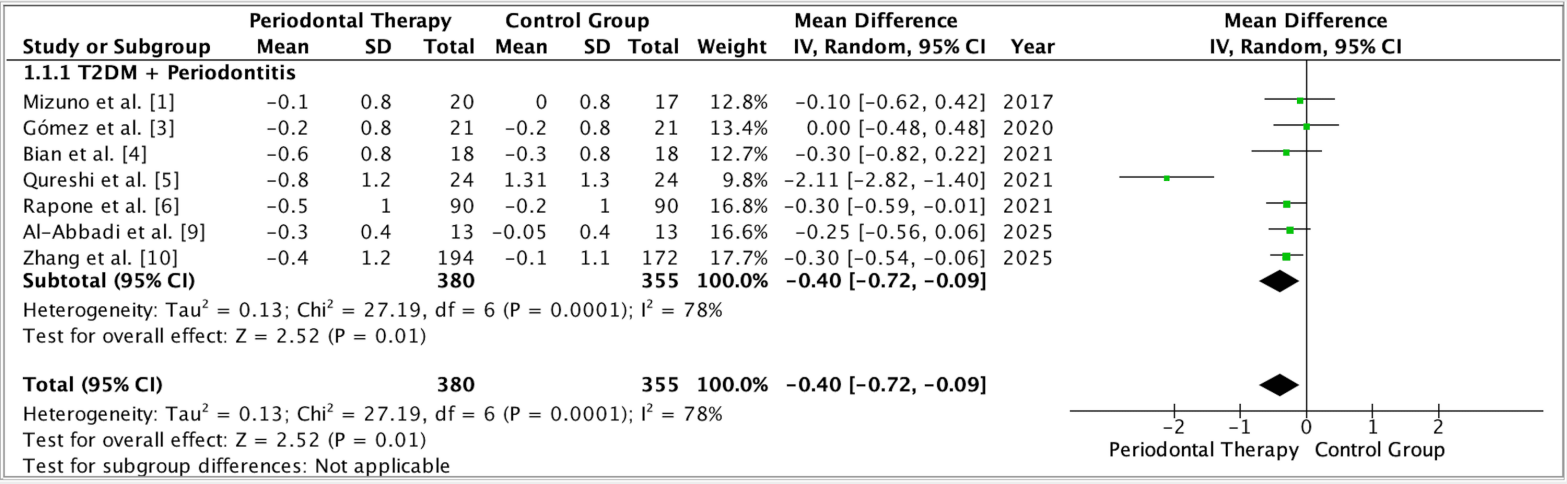
Forest plot (HbA1c, % at ≈3 months). Pooled mean difference (Intervention − Comparator) in ΔHbA1c (%) using a fixed-effect model across adults with type 2 diabetes mellitus (T2DM) and periodontitis. Overall effect favors intervention: MD −0.38% (95% CI −0.52 to −0.25). Abbreviations: HbA1c, glycated hemoglobin; MD, mean difference.

**Figure 3:**
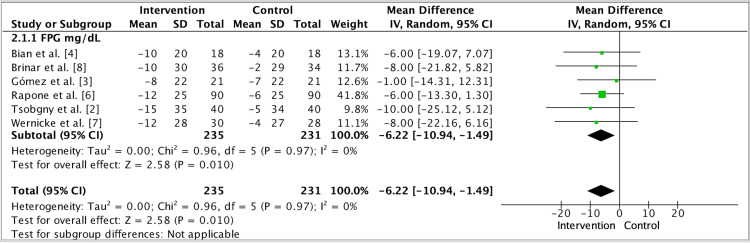
Intervention comparing fasting plasma glucose vs control group Random-effects meta-analysis of change in fasting plasma glucose (mg/dL) comparing Intervention vs Comparator. Pooled effect favors Intervention: MD −6.22 mg/dL (95% CI −10.94 to −1.49). Abbreviations: FPG, fasting plasma glucose; MD, mean difference.

**Figure 4:**
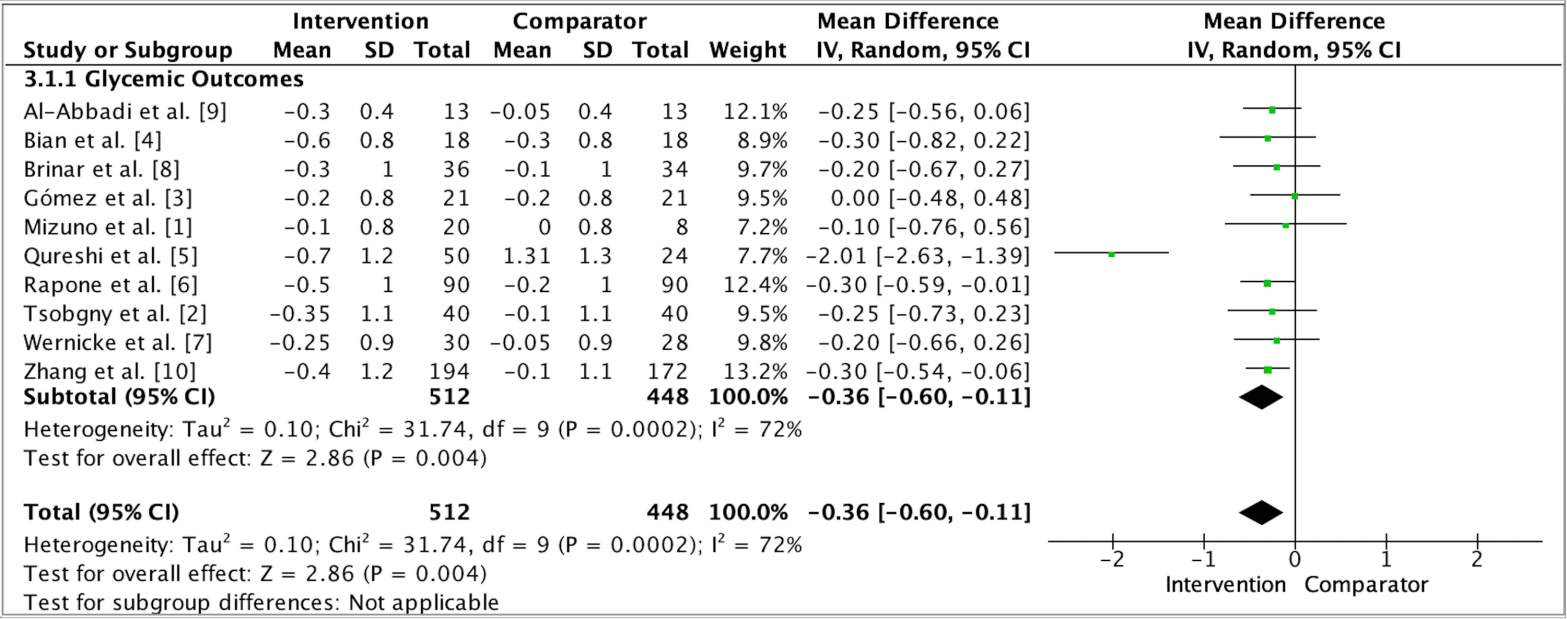
Subgroup analysis: Intervention comparing HbA1c outcomes versus control group. Random-effects meta-analysis of HbA1c, including all available trials under a single subgroup. Overall effect favors Intervention: MD −0.35% (95% CI −0.60 to −0.11). Abbreviations: HbA1c, glycated hemoglobin; MD, mean difference.

**Figure 5:**
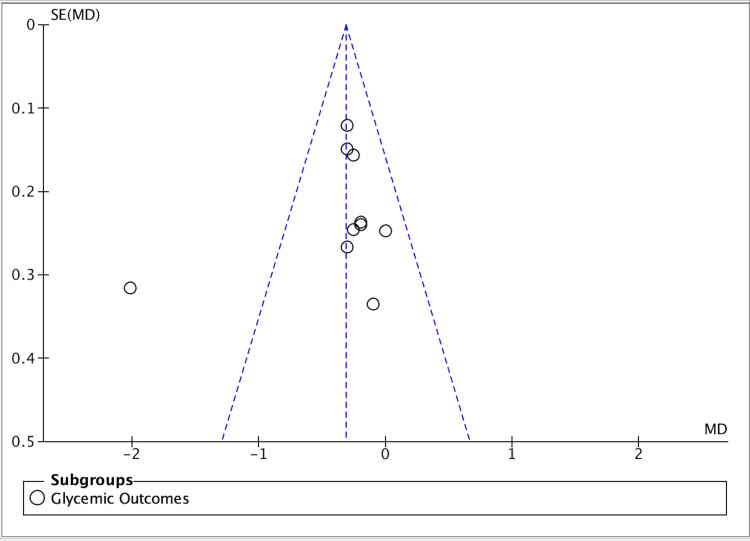
Funnel plot. Assessment of publication bias Funnel plot of study precision vs effect size for the HbA1c outcome; the scatter shows no marked small-study asymmetry on visual inspection, suggesting low risk of publication bias. Abbreviations: HbA1c, glycated hemoglobin

Heterogeneity across trials was expected. Baseline metabolic control ranged from poor to adequate; follow-up windows varied (primarily three to six months), and analytic choices (change scores vs final values, site- vs subject-level summaries) differed [1]. Several trials used delayed-treatment or hygiene-advice controls, others routine care comparators, and a large cluster trial tested community-level integration - differences that influence both internal and external validity [6,10]. Despite this diversity, the direction of effect favored intervention for periodontal outcomes across settings, and forest plot synthesis of the pooled dataset preserved this signal (Figures 2-4). Funnel plot inspection did not reveal substantial asymmetry, although with about ten studies, small-study effects cannot be excluded confidently (Figure 5).

Mechanistically, reductions in periodontal inflammation may ameliorate systemic inflammatory tone (e.g., CRP and oxidative stress), easing peripheral insulin resistance and improving glycemic variability over time [1]. Trials registering significant HbA1c decline often combined intensive NSPT with strong behavioral supports or enrolled participants with higher baseline HbA1c, in whom absolute change potential is greater [2]. Conversely, neutral glycemic findings despite clear periodontal gains likely reflect ceiling effects from optimized diabetes pharmacotherapy, short follow-up insufficient to capture HbA1c turnover, or dilution from lifestyle confounders outside dental care [6].

Implementation signals emerged from programmatic studies. Community-embedded, integrated curricula improved glycemic control rates and oral symptoms beyond routine care, arguing for policy models that tie periodontal maintenance to diabetes self-management education, peer support, and primary care coordination [10]. At the individual level, structured physical activity reduced gingival inflammation and improved HbA1c, reinforcing the notion that periodontal health is co-produced with systemic lifestyle interventions rather than achieved by instrumentation alone [7]. These observations support a multidisciplinary approach: NSPT as the procedural core, coupled with diabetes education, activity counseling, and pharmacologic optimization [7].

Limitations temper inferences. Masking is challenging in periodontal trials; supervision intensity and maintenance schedules vary, and some studies rely on short windows that may under-estimate longer-term metabolic change [1]. Differences in adjunct protocols (drug choice, dosing, aPDT parameters, hyaluronic-acid formulations) and outcome definitions add methodological noise [8,9]. A subset of studies used delayed-treatment rather than parallel minimal-care controls, and adherence verification outside clinic visits remains imperfect, introducing potential performance and detection biases [5,6]. Nonetheless, sensitivity analyses anchored by the pooled forest plots retained the periodontal benefit signal, while glycemic effects, though smaller, were consistently directionally favorable [1] (Figures 2-4).

Clinically, the findings justify integrating periodontal care into routine diabetes management. For most adults with T2DM and periodontitis, high-quality NSPT with standardized self-care instruction should be first-line; adjuncts may be reserved for specific indications (e.g., severe sites, limited response, regenerative aims) with attention to stewardship and patient preferences [3,9]. Systems-level interventions that embed periodontal counseling within diabetes programs may amplify metabolic benefits and improve scalability and equity [10]. Future trials should extend follow-up beyond six to 12 months, standardize core outcomes, and stratify by baseline HbA1c and inflammatory phenotype to clarify who benefits most and when [4]. Cost-effectiveness analyses - especially for community-integrated models and non-antibiotic adjuncts - are needed to inform coverage decisions and maintenance intervals [8,10].

In sum, contemporary evidence indicates that periodontal therapy reliably improves periodontal health and likely confers small, clinically meaningful improvements in glycemic control when delivered within supportive behavioral frameworks [1,2]. Priorities ahead include longer-term durability studies, judicious adjunct selection, and implementation research that binds dental and diabetes care into cohesive, patient-centered pathways [5,6,10].

Limitations

This review has limitations. Some of them included trials showed at least some risk of bias, mainly due to unclear allocation concealment, lack of blinding of operators or outcome assessors, and incomplete methodological reporting. Sample sizes were modest, with follow-up typically limited to three to six months, which restricts inference about long-term metabolic control and hard clinical outcomes. There was also notable clinical heterogeneity in periodontal protocols, baseline HbA1c levels, and background antidiabetic therapy, which may have influenced the pooled estimates. In addition, several potentially relevant outcomes (inflammatory markers, oxidative stress indices, quality of life, and costs) were reported sparsely or with non-standardized measures, and the restriction to English-language studies with extractable numeric data raises the possibility of language and publication bias.

Recommendations

Future research should focus on larger, rigorously designed RCTs with proper allocation concealment, blinded outcome assessment, and prospective registration to strengthen confidence in the metabolic findings. Periodontal interventions and maintenance schedules should be standardized to improve comparability and pooling across studies, with follow-up extended beyond three to six months to assess the durability of HbA1c and fasting plasma glucose improvements. In addition, trials should incorporate hard clinical endpoints (such as cardiovascular events, hospitalizations, and progression of diabetic complications) and systemic inflammatory markers, rather than relying solely on surrogate outcomes, to better define the overall impact of periodontal therapy on health in individuals with T2DM.

## Conclusions

Active periodontal care - especially high-quality NSPT with hygiene instruction - consistently outperforms minimal/routine care for PPD, CAL, BOP, and plaque. Adjuncts (systemic antibiotics, aPDT, and topical hyaluronic acid) can add context-dependent benefits, but meticulous mechanical therapy is foundational. Glycemic improvements are modest yet generally favorable, particularly when paired with behavioral/self-management supports. Given heterogeneity and short follow-up, longer standardized trials are needed to clarify HbA1c durability and identify subgroups most likely to benefit and optimal maintenance schedules.
